# Comparison of the effects of inhalational and total intravenous anesthesia on quality of recovery in patients undergoing endoscopic transsphenoidal pituitary surgery: a randomized controlled trial

**DOI:** 10.7150/ijms.72758

**Published:** 2022-06-13

**Authors:** Do-Hyeong Kim, Kyeong Tae Min, Eui Hyun Kim, Young Seo Choi, Seung Ho Choi

**Affiliations:** 1 Department of Anesthesiology and Pain Medicine, Anesthesia and Pain Research Institute, Yonsei University College of Medicine, Seoul, Republic of Korea.; 2 Department of Neurosurgery, Pituitary Tumor Center, Yonsei University College of Medicine, Seoul, Republic of Korea.

**Keywords:** inhalational anesthesia, intravenous anesthesia, postoperative recovery, pituitary surgery

## Abstract

**Background:** Endoscopic transsphenoidal pituitary surgery has shown promising results. However, fast and high-quality recovery after this procedure remains a challenge for neuroanesthesiologists. This study aimed to compare the quality of recovery after transsphenoidal pituitary surgery between patients who received inhalational anesthesia with sevoflurane and patients who received propofol-based total intravenous anesthesia (TIVA).

**Methods:** Eighty-two patients undergoing transsphenoidal pituitary surgery were randomized to receive either sevoflurane inhalation with manual infusion of remifentanil (sevoflurane group) or effect-site target-controlled infusion of propofol and remifentanil (TIVA group). The primary outcome was the 40-item Quality of Recovery (QoR-40) score on postoperative day 1. The QoR-40 questionnaire was completed by patients the day before surgery and on postoperative days 1 and 2. Emergence agitation and recovery characteristics were also assessed.

**Results:** There were no significant differences between the groups in the global QoR-40 scores on both postoperative days 1 and 2 (difference -8.7, 95% CI -18.0 to 0.7, and *P* = 0.204; -3.6, 95% CI -13.0 to 5.8, and *P* > 0.999, respectively). The time to verbal response and time to extubation were significantly shorter in the sevoflurane group than in the TIVA group (*P* < 0.001 and *P* < 0.001, respectively). However, the incidence of emergence agitation was lower in the TIVA group than in the sevoflurane group (*P* < 0.001).

**Conclusions:** Both inhalational anesthesia with sevoflurane and propofol-based TIVA were appropriate anesthetic techniques for patients undergoing endoscopic transsphenoidal pituitary surgery in terms of the quality of recovery up to 2 days postoperatively. Rapid emergence was observed in the sevoflurane group, while smooth emergence was observed in the TIVA group.

## Introduction

The transsphenoidal approach for resection of pituitary tumors has evolved over the past decades and is currently a mainstream surgical technique 1, 2. Endoscopic transsphenoidal surgery offers some advantages such as minimal invasiveness, improved visualization of the surgical field, and lower incidence of complications, which may lead to lower morbidity and mortality rates 3-5. However, this procedure causes intense noxious stimuli at various stages of the surgery, which result in difficulties in maintaining intraoperative hemodynamic stability 6. Furthermore, rapid and smooth emergence is desirable in patients undergoing this type of surgery because the immediate postoperative use of nasal packing requires conscious mouth breathing and may cause difficulties in airway management. Adequate emergence also lowers the risk of surgical complications such as cerebrospinal fluid rhinorrhea due to coughing and enables a prompt neurological examination 7-10. Moreover, there is an increasing demand for enhanced postoperative recovery after endoscopic pituitary surgery that is not limited to the immediate postoperative period 11. Therefore, high quality recovery after endoscopic transsphenoidal surgery remains a challenge for neuroanesthesiologists.

The findings of previous research comparing the effects of inhalational anesthesia and propofol-based total intravenous anesthesia (TIVA), the two most commonly used techniques for general anesthesia, on patient postoperative recovery, are inconsistent as different results have been demonstrated according to the type of surgery, patient population, and recovery outcomes 12. Some studies have compared inhalational anesthesia and TIVA in patients undergoing endoscopic transsphenoidal pituitary surgery. However, these studies focused on intraoperative hemodynamic parameters and fragmentary measures of recovery profile during emergence from anesthesia and the immediate postoperative period 9, 13. To date, there is a lack of research performing a comprehensive assessment of recovery quality after transsphenoidal surgery.

This study aimed to compare the effects of inhalational anesthesia with sevoflurane and propofol-based TIVA on the quality of recovery assessed by the 40-item Quality of Recovery (QoR-40) questionnaire, a validated multidimensional assessment tool 14, 15, in patients undergoing endoscopic transsphenoidal pituitary surgery. We hypothesized that TIVA would provide better patient-perceived quality of recovery than inhalational anesthesia in these patients. Moreover, we compared other recovery profiles during the emergence period and postanesthesia care unit (PACU) stay.

## Materials and Methods

This randomized controlled trial was performed between June 2016 and June 2018 at Severance Hospital, Yonsei University Health System, Seoul, Korea, in accordance with the tenets of the Declaration of Helsinki. The study protocol was approved by the Institutional Review Board and Hospital Research Ethics Committee of Severance Hospital, Yonsei University Health System (#4-2016-0344) on June 16, 2016 and registered at ClinicalTrials.gov (NCT02813044) on June 24, 2016. Written informed consent was obtained from all patients. Patients aged 19 years or older with an American Society of Anesthesiologists physical status I or II, who were scheduled to undergo transsphenoidal surgery for pituitary tumor, were enrolled in this study. Exclusion criteria were as follows: history of allergy to any study drug; left ventricular ejection fraction <55%; third-degree or second-degree atrioventricular block; history of myocardial infarction; stroke or cardiac surgery within the previous 1 year; severe neurological disease; and use of sedatives, opioids or sleep-inducing drugs.

Enrolled patients were randomly allocated to receive either inhalational anesthesia with sevoflurane (sevoflurane group) or propofol-based TIVA (TIVA group) on the day of surgery in a 1:1 ratio, according to a computer-generated randomization sequence by an investigator not involved in patient care or perioperative assessment. Because of significant differences between the anesthetic techniques, the attending anesthesiologists could not be blinded to the group assignment. However, the anesthesiologists were not involved in the study. Patients and investigators in charge of the perioperative assessment or data analysis were blinded to the method of anesthesia during the study period.

On arrival in the operating room, standard monitoring and measurement of the bispectral index were commenced. Glycopyrrolate 0.1 mg was administered to the patients immediately before anesthesia induction. In the sevoflurane group, anesthesia was induced with a bolus administration of 4-6 mg/kg of pentothal sodium and 1-2 μg/kg of remifentanil, and then maintained with inhalation of sevoflurane at a 0.8-1.0 age-adjusted minimal alveolar concentration and infusion of 0.02-0.2 μg/kg/min of remifentanil. In the TIVA group, anesthesia was induced and maintained with an effect-site target-controlled infusion (TCI) of propofol (2-6 μg/ml) and remifentanil (2-6 ng/ml) using a TCI pump (Orchestra Base Primea, Fresenius Vial, Brezins, France) based on the Marsh and Minto model 16, 17, respectively. In both groups, rocuronium 0.9 mg/kg was administered before intubation; then, patients were mechanically ventilated using constant-flow volume-controlled ventilation with an air/oxygen mixture (fraction of inspired oxygen 0.5). The tidal volume was 6-8 ml/kg predicted body weight; the respiratory rate was adjusted to maintain an end-tidal carbon dioxide tension of 35-38 mmHg. A radial artery catheter was placed for continuous arterial pressure monitoring. The concentrations of sevoflurane and propofol were adjusted to maintain anesthetic depth, aiming for a bispectral index of 40-60. Remifentanil was also adjusted to maintain the mean arterial blood pressure (MAP) and heart rate (HR) within 80%-120% of preoperative values. Hypotension (MAP <80% of baseline) persisting for 5 min was treated with normal saline boluses and, if hypotension persisted, ephedrine, phenylephrine, or norepinephrine were administered at the discretion of the attending anesthesiologist. Bradycardia (HR <40/min) was treated with atropine 0.5 mg. At 30 min before the end of surgery, ramosetron 0.3 mg and nefopam 40 mg were administered for antiemetic prophylaxis and postoperative analgesia, respectively. Following completion of the procedure, the neuromuscular block was reversed with neostigmine and glycopyrrolate after confirming the return of neuromuscular function using train-of-four peripheral nerve stimulation. At this time, all anesthetics (sevoflurane, propofol, and remifentanil) were discontinued and 100% oxygen was administered at a flow rate of 8 l/min. No stimulation was given to the patients except for repetitive verbal requests to open their eyes. Extubation was performed when patients were able to obey verbal requests and were breathing adequately. All patients were transferred to the PACU.

In the PACU, postoperative pain was assessed using an 11-point numeric rating scale (NRS: 0 = no pain, 10 = worst imaginable pain). Intravenous fentanyl 1.0 μg/kg was administered as a rescue analgesic when the pain score at rest was ≥ 4 or on patient request. Postoperative nausea and vomiting on a scale 0-3 (none, mild, moderate, and severe) were also assessed. If severe nausea or vomiting occurred, metoclopramide 10 mg was administered. In the ward, all patients received nefopam 20 mg intravenously every 12 hours up to POD 2 and ibuprofen 400 mg orally every 8 hours until discharge to maintain an NRS pain score of <4. However, if the patients reported a persistent NRS pain score of ≥4 or upon patient request, intravenous or intramuscular rescue tramadol 25-50 mg was administered. For antiemetic prophylaxis, intravenous granisetron 1 mg was administered every 12 hours up to POD 2. The patients were treated with 10 mg of metoclopramide if severe nausea or vomiting occurred.

The primary outcome was the global QoR-40 score on the first postoperative day (POD). The QoR-40 contains 40 items assessing five recovery domains: emotional status (nine items), physical comfort (12 items), psychological support (seven items), physical independence (five items), and pain (seven items) 14, 18. Each item is graded on a five-point Likert scale, and global QoR-40 scores range from 40 to 200. The patients completed the questionnaire 1 day preoperatively and on PODs 1 and 2. The secondary outcomes included time to verbal response, time to extubation, and emergence agitation. Time to verbal response and time to extubation were defined as the time from the cessation of anesthetics to the patient's response to verbal command and to tracheal extubation, respectively. The period from the end of surgery to 2 min after extubation was defined as the emergence period. During the emergence period, the patient's maximum agitation score was recorded using the Riker sedation-agitation scale: 1 = minimal or no response to noxious stimuli; 2 = arouse to physical stimuli but does not communicate; 3 = difficult to arouse but awakens to verbal stimuli or gentle shaking; 4 = calm and follows commands; 5 = anxious or physically agitated and calms to verbal instructions; 6 = requiring restraint and frequent verbal reminding of limits; 7 = pulling at tracheal tube, trying to remove catheters or striking at staff 19. Emergence agitation was defined as a sedation-agitation scale score of ≥ 5 20. In addition, the grade of cough during the emergence period was assessed using a 4-point scale: 0 = no cough; 1 = single cough; 2 = persistent cough lasting < 5 s; 3 = persistent cough lasting ≥ 5 s or bucking 20. The following perioperative data were also collected: MAP and HR (at baseline before anesthetic induction, 10 and 30 min after the start of surgery, at cessation of the main anesthetics, at tracheal extubation, and 10 and 30 min after PACU admission), pain scores and nausea and vomiting scores in the PACU, length of hospital stay, postoperative complications such as diabetes insipidus. All outcome and perioperative data were collected by an investigator blinded to the group allocation.

### Statistical Analysis

The global QoR-40 score on POD 1 has been reported as 174 ± 16.2 21. A difference in global QoR-40 score of 10 or more between groups was considered clinically significant 22, 23. To obtain a power of 0.80 (1-β) with an α of 0.05, the calculated sample size was 37 patients per group. To allow for a dropout rate of 10%, 41 patients per group were required.

Normality of the data distribution was assessed using the Shapiro-Wilk test. Continuous variables were analyzed using the independent t-test or Mann-Whitney U-test. Categorical variables were analyzed by the χ2 test or Fisher's exact test. Values are presented as mean ± standard deviation, median (interquartile range), or number of patients (proportion) as appropriate. Repeated measures variables including QoR-40 scores, MAP, and HR were assessed using a linear mixed model with patient indicator as a random effect, and group, time, and group-by-time interaction as fixed effects. An autoregressive covariance structure was used. These linear mixed model analyses were followed by post hoc test with a Bonferroni correction to control the familywise error rate. Additionally, logistic regression analysis was performed using the Enter method and the backward stepwise method to assess possible factors affecting a decrease of the minimal clinically important difference (6.3) or the global QoR-40 score on POD 1 from the preoperative QoR-40 score 24. Based on the previous report 25 and our results about intraoperative data, the type of anesthetic technique and sex, age, diagnosis, and total dose of remifentanil used, after checking for multicollinearity, were included in the analysis. Model fit was assessed with the Hosmer-Lemeshow goodness-of-fit test. All analyses were performed using R version 4.0.3 (The R Foundation for Statistical Computing, Vienna, Austria), MedCalc version 20 (MedCalc, Ostend, Belgium), and Statistical Package for the Social Sciences version 25 (IBM Corp., Armonk, NY, USA). *P* < 0.05 was considered statistically significant.

## Results

**Figure 1:**
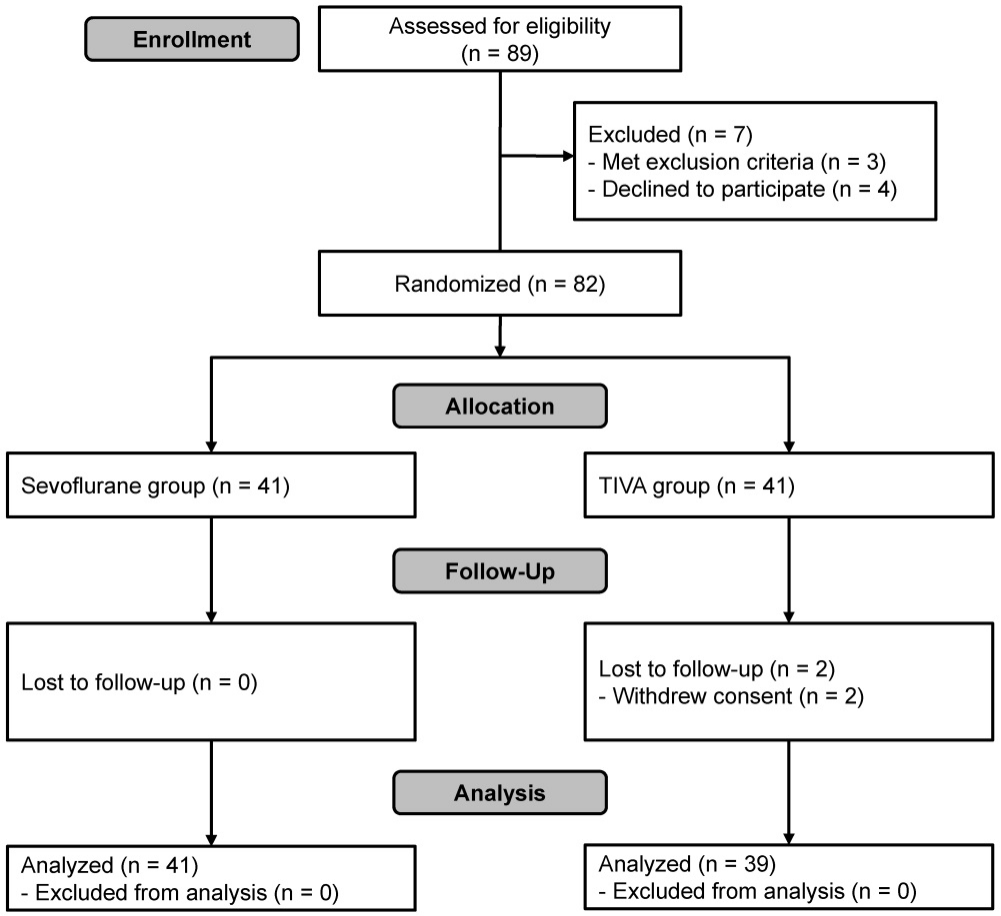
Flowchart of patient selection. TIVA, total intravenous anesthesia.

A total of 89 patients were assessed for eligibility. Of these, seven were excluded because of cognitive impairment (n = 1), history of myocardial infarction (n = 1), intranasal ethmoidectomy scheduled to be performed simultaneously due to chronic sinusitis (n = 1) or refusal to participate (n = 4). Thus, 82 subjects were enrolled in this study and randomized. Two were excluded from the final analysis because of refusal to participate during follow-up (n = 2, TIVA group). In total, 80 patients completed the study (Figure 1). Patient characteristics and details of surgery were not significantly different between the groups except for the total dose of remifentanil used, which was significantly greater in the TIVA group than in the sevoflurane group (Table 1).

The QoR-40 scores are shown in Table 2. The group-by-time interaction for the comparison of global QoR-40 scores between the sevoflurane group and the TIVA group was not significant (*P* = 0.923). The global QoR-40 scores on both PODs 1 and 2 were not significantly different between the groups (difference -8.7, 95% CI -18.0 to 0.7, and *P* = 0.204; -3.6, 95% CI -13.0 to 5.8, and *P* > 0.999, respectively). Among the five dimensions, the group-by-time interaction on the physical independence dimension was only statistically significant between the 2 groups over time (*P* = 0.044). However, the scores of the physical independence dimension on both PODs 1 and 2 were comparable between the groups. Table 3 shows the adjusted odds ratio and the *P*-value of each variable for a decrease of 6.3 or more in the global QoR-40 score on POD 1 from the preoperative QoR-40 score. After backward stepwise selection, only the total dose of remifentanil used was independently associated with a decrease of minimal clinically important difference or more (odds ratio 0.521, 95% confidence interval 0.285-0.950, *P* = 0.033).

During the emergence period, time to verbal response and time to extubation were significantly shorter in the sevoflurane group than in the TIVA group. However, the incidence of emergence agitation was lower in the TIVA group than that in the sevoflurane group, and grade of cough was also lower in the TIVA group (Table 4). During the PACU stay, maximal NRS pain score was lower in the TIVA group than that in the sevoflurane group; however the use of rescue analgesics was not different between the two groups. The use of antiemetics in the PACU was significantly lower in the TIVA group (Table 4).

**Figure 2:**
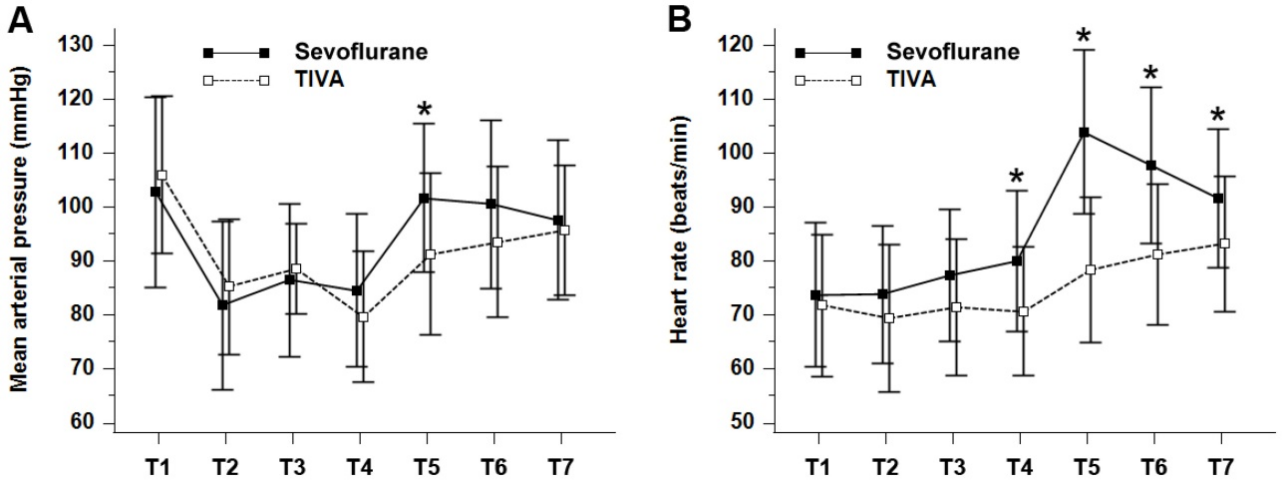
Perioperative (**A**) mean arterial pressure (mmHg) and (**B**) heart rate (beats/min). Values are presented as mean ± standard deviation. Mean arterial pressure (P_Group × Time_ = 0.024) and heart rate (P_Group × Time_ = 0.0002) were significantly lower in the TIVA group compared with the sevoflurane group over time. TIVA, total intravenous anesthesia; T1, at baseline before anesthetic induction; T2, 10 min after the start of operation; T3, 30 min after the start of operation; T4, at cessation of main anesthetics; T5, at tracheal extubation; T6, 10 min after postanesthesia care unit admission; T7, 30 min after postanesthesia care unit admission. *P < 0.05 compared with TIVA group (Bonferroni corrected).

Perioperative MAP and HR are shown in Figure 2. The MAP (*P* = 0.024) and HR (*P* = 0.0002) were significantly higher in the sevoflurane group when the values for all the time points during the perioperative period were combined. In particular, both MAP and HR were significantly higher in the sevoflurane group than in the TIVA group at tracheal extubation (in the emergence period), and HR was significantly higher in the sevoflurane group at each time point during the PACU stay.

The incidences of postoperative complications such as diabetes insipidus were comparable between the groups (Table 5). There was no difference between the two groups in length of hospital stay.

## Discussion

Patients with brain tumors may have vulnerable central nervous systems and may be more susceptible to anesthetic and surgical insults than the general surgical population thereby hindering postoperative recovery. Moreover, the endoscopic transsphenoidal approach causes difficulties in anesthetic care and airway management, leading to worse recovery profiles, particularly in the immediate postoperative period 9. Therefore, it is important to find an adequate anesthetic regimen that can provide high quality recovery after transsphenoidal pituitary surgery. In the present study, the choice between inhalational anesthesia and TIVA, an essential part of anesthetic strategies, did not affect global QoR-40 scores in patients undergoing transsphenoidal pituitary surgery. During the immediate postoperative period, the sevoflurane group had rapid emergence while the TIVA group had smooth emergence.

The modulation of inflammatory and stress responses stimulated by anesthesia and surgery is associated with an improvement in the quality of recovery 26. Compared with inhalational anesthetics, propofol was known to have anti-inflammatory and antioxidant effects 27, 28. Propofol has been reported to inhibit the release of pro-inflammatory cytokines and to reduce the production of lipopolysaccharide-induced reactive oxygen species via inhibition of inflammatory factors 29, 30. In a previous study, propofol was associated with significantly higher anti-inflammatory cytokine levels than sevoflurane in patients undergoing craniotomy 31. In another study postoperative quality of recovery in patients undergoing endoscopic sinus surgery was better with propofol-based TIVA than with desflurane anesthesia 32. However, in the present study, despite differences in the modulation of inflammatory and stress responses between propofol and inhalational anesthetics, global QoR-40 scores after endoscopic transsphenoidal pituitary surgery were not influenced by the type of general anesthesia. The burden of surgical stress and inflammation may vary depending on the type of surgery and patient population, which may explain our findings. Moreover, there were some studies reporting beneficial anti-inflammatory effects of inhalational anesthetics 33, 34. Therefore, the differences in anti-inflammatory effects between propofol and inhalational anesthetics may also vary.

Consistent with earlier studies, both time to verbal response and time to extubation were shorter in the sevoflurane group than in the TIVA group 9, 13. However, this study found that TIVA was better than sevoflurane anesthesia with regard to agitation and coughing during emergence, based on evaluation using graded scales unlike previous studies. This incidence of emergence agitation in the sevoflurane group was similar to that reported in otorhinolaryngology procedures 35. TIVA also demonstrated better antiemetic effects during the PACU stay, a well-known advantage of propofol-based TIVA over inhalational anesthesia 36. However, it seems that the benefits of each anesthetic method could not lead to an improvement in the quality of recovery as well as a decrease in the length of hospital stay. A recent review article on perioperative anesthetic management during transsphenoidal pituitary surgery did not recommend a particular anesthetic technique over another 8. Therefore, the selection of the most appropriate anesthetic method should be case-specific, according to the condition and surgical situation of the individual patient.

In this study, although the use of rescue analgesics was not different, the maximal pain score during the PACU stay was lower in the TIVA group than that in the sevoflurane group. The type of anesthesia has been known as a factor that influences postoperative pain 36. Several studies have shown propofol maintenance with fentanyl or remifentanil to be associated with less postoperative pain than inhalational anesthesia 37, 38. Propofol not only has an intrinsic analgesic effect but also can delay and weaken remifentanil-induced hyperalgesia via its antagonistic effect on N-methyl-D-aspartate receptors 39, 40. On the contrary, according to a previous study, inhaled anesthetics tend to produce hyperalgesia at minimum alveolar concentrations of 0.1, which may increase pain perception during recovery from anesthesia 41. However, a recent meta-analysis did not demonstrate a significant difference in postoperative pain intensity at 30 min after surgery between propofol anesthesia and inhalational anesthesia 42.

There is a growing interest in enhanced recovery after surgery (ERAS), and ERAS protocols are increasingly being investigated in the context of different surgical specialties including neurosurgery 43. In a recent study aimed at developing and assessing the ERAS protocol for endoscopic transsphenoidal pituitary surgery, TIVA was included in the protocol as the main anesthetic technique 11. However, this choice was based on a previously published report concluding that the risk for postoperative nausea and vomiting and time in the PACU were lower with propofol than with inhalational agents in ambulatory and inpatient surgery 36. The results of our study could aid in establishing enhanced recovery protocols for endoscopic transsphenoidal pituitary surgery.

This study has several limitations. First, the patients in our study were relatively healthy, which limits the generalizability of our results. However, this is consistent with the observation that patients undergoing cranial surgery generally have a good health status preoperatively 15. Second, although anesthetic techniques can affect perioperative neuroendocrine functions, which may be a confounding factor, we did not assess the neuroendocrine stress response. However, recent retrospective studies have reported that the effect of anesthetic techniques on neuroendocrine function may be limited and may disappear shortly after the end of anesthesia 44, 45. Third, we did not conduct long-term postoperative follow-ups. While it is known that there is also a relationship between the quality of recovery in the days and weeks after surgery 46, further studies comparing the effects of anesthetic methods on recovery trajectory over time are needed in patients undergoing transsphenoidal pituitary surgery. Fourth, the difference in the total dose of remifentanil used between the groups may have affected postoperative QoR-40 scores. However, the purpose of this study was not to compare sevoflurane and propofol but to compare sevoflurane inhalation with a manual infusion of remifentanil and effect-site target-controlled infusion of propofol and remifentanil. Further studies are needed to address this issue.

In conclusion, overall QoR-40 scores after endoscopic transsphenoidal pituitary surgery were not significantly different up to POD 2 between patients receiving sevoflurane anesthesia and those receiving propofol-based TIVA. The sevoflurane anesthesia resulted in a faster emergence, while the propofol-based TIVA lowered the incidence of agitation and the severity of cough during emergence.

## Figures and Tables

**Table 1:** Patient characteristics and details of surgery

	Sevoflurane group (n = 41)	TIVA group (n = 39)	*P*
Female	25 (61.0)	22 (56.4)	0.851
Age (y)	44.0 (36.0-55.0)	52.0 (41.0-58.5)	0.110
Height (cm)	166.0 ± 9.0	164.5 ± 8.7	0.425
Weight (kg)	68.0 ± 11.0	67.7 ± 11.6	0.927
BMI (kg/m^2^)	24.6 ± 3.1	24.9 ± 2.7	0.630
ASA physical status (I/II)	28/13	23/16	0.526
**Comorbidity**			
Hypertension	9 (22.0)	9 (23.1)	>0.999
Diabetes mellitus	6 (14.6)	7 (17.9)	0.922
Ischemic heart disease	2 (4.9)	0 (0.0)	0.496
**Tumor type**			0.134
Non-functioning adenoma	14 (34.1)	22 (56.4)	
GH-secreting adenoma	12 (29.3)	8 (20.5)	
ACTH-secreting adenoma	1 (2.4)	2 (5.1)	
Prolactine-secreting adenoma	11 (26.8)	3 (7.7)	
FSH-secreting adenoma	0 (0.0)	1 (2.6)	
Rathke's cleft cyst	3 (7.3)	3 (7.7)	
**Tumor size**			0.198
≤ 1cm	11 (26.8)	5 (12.8)	
> 1cm	30 (73.2)	34 (87.2)	
Carvernous sinus invasion	9 (22.0)	15 (38.5)	0.172
Intraoperative CSF leakage	11 (26.8%)	18 (47.4%)	0.097
Total blood loss (ml)	150.0 (100.0-220.0)	150.0 (67.5-210.0)	0.745
Patients received vasopressors	19 (46.3)	16 (41.0)	0.800
Total dose of propofol used (mg)	-	2036.0 (1723.0-2729.5)	-
Total dose of remifentanil used (μg)	1142.0 (900.0-1400.0)	2015.0 (1629.0-2739.0)	<0.001
Duration of surgery (min)	164.7 ± 47.6	169.1 ± 62.2	0.720
Duration of anesthesia (min)	246.6 ± 55.3	250.4 ± 71.5	0.791

Data are presented as mean ± standard deviation, median (interquartile range), or number of patients (%). ACTH, adrenocorticotropic hormone; ASA, American Society of Anesthesiologists; BMI, body mass index; CSF, cerebrospinal fluid; FSH, follicle stimulating hormone; GH, growth hormone; TIVA, total intravenous anesthesia.

**Table 2:** Preoperative and postoperative QoR-40 scores

	Sevoflurane group (n = 41)	TIVA group (n = 39)	Difference (95% CI)	*P*_Group×Time_*	Adjusted *P*†
**Global QoR-40**				0.923	
Preoperative	179.4 (1.7)	183.1 (1.6)	-3.6 (-8.3 to 1.0)		0.381
POD 1	157.3 (3.3)	166.0 (3.3)	-8.7 (-18.0 to 0.7)		0.204
POD 2	166.6 (3.2)	170.2 (3.5)	-3.6 (-13.0 to 5.8)		>0.999
**Emotional status**				0.451	
Preoperative	37.0 (0.8)	39.3 (0.7)	-2.3 (-4.4 to -0.3)		0.084
POD 1	37.4 (0.9)	38.6 (0.8)	-1.3 (-3.7 to 1.2)		0.915
POD 2	38.3 (0.9)	39.8 (0.9)	-1.5 (-4.1- to 1.1)		0.741
**Physical comfort**				0.668	
Preoperative	53.8 (0.8)	54.6 (0.8)	-0.8 (-2.9 to 1.3)		>0.999
POD 1	44.5 (1.1)	47.0 (1.3)	-2.5 (-5.9 to 0.9)		0.426
POD 2	47.6 (1.3)	47.7 (1.5)	-0.1 (-4.1 to 3.9)		>0.999
**Psychological support**				0.882	
Preoperative	31.9 (0.4)	32.4 (0.5)	-0.6 (-1.8 to 0.7)		>0.999
POD 1	29.6 (0.6)	31.5 (0.6)	-1.8 (-3.5 to -0.1)		0.111
POD 2	31.1 (0.7)	31.6 (0.8)	-0.5 (-2.8 to 1.7)		>0.999
**Physical independence**				0.044	
Preoperative	24.5 (0.2)	24.5 (0.2)	0.0 (-0.6 to 0.6)		>0.999
POD 1	19.9 (0.8)	20.7 (0.8)	-0.8 (-3.0 to 1.4)		>0.999
POD 2	21.5 (0.7)	23.0 (0.6)	-1.4 (-3.2 to 0.2)		0.273
**Pain**				0.715	
Preoperative	32.3 (0.5)	32.3 (0.5)	0.0 (-1.3 to 1.4)		>0.999
POD 1	25.9 (0.9)	28.3 (0.7)	-2.4 (-4.7 to -0.1)		0.129
POD 2	28.7 (0.9)	29.0 (0.9)	-0.2 (-2.7 to 2.2)		>0.999

Data are presented as mean (standard error). CI, confidence interval; POD, postoperative day; QoR-40, 40-item Quality of Recovery questionnaire; TIVA, total intravenous anesthesia. *P value of the group-by-time interaction in the linear mixed model. †*P* value was corrected using the Bonferroni correction for multiple comparisons.

**Table 3:** Logistic regression analysis* for possible factors associated with a decrease of 6.3 (minimal clinically important difference) or more in the global QoR-40 score on the first postoperative day compared to the preoperative QoR-40 score

	Odds ratio (95% confidence interval)	*P*
TIVA group	1.15 (0.30-4.36)	0.832
Female sex	1.04 (0.36-2.97)	0.941
Age†	0.99 (0.95-1.03)	0.656
Non-functioning adenoma	0.88 (0.31-2.51)	0.810
Total dose of remifentanil used ‡	0.48 (0.21-1.11)	0.086

QoR-40, 40-item Quality of Recovery questionnaire; TIVA, total intravenous anesthesia. *Enter method, Hosmer-Lemeshow χ^2^, 8.050, *P* = 0.429. †Per 1-year increase. ‡Per 1-mg increase.

**Table 4:** Recovery characteristics

	Sevoflurane group (n = 41)	TIVA group (n = 39)	*P*
Emergence*			
Time to verbal response (min)†	7.5 (6.0-10.1)	14.4 (11.1-17.5)	<0.001
Time to extubation (min)‡	8.5 (6.3-10.2)	15.9 (11.5-18.1)	<0.001
Sedation-agitation scale score	4.0 (3.0-5.0)	4.0 (3.0-4.0)	0.001
Emergence agitation¶	17 (43.6)	0 (0.0)	<0.001
Grade of cough§	1.0 (0.0-2.0)	0.0 (0.0-1.0)	0.002
**PACU**			
Maximal NRS pain score	4.0 (2.0-5.0)	2.0 (0.0-4.0)	0.020
Use of analgesics at PACU	9 (22.0)	4 (10.3)	0.265
Maximal PONV score**	0.0 (0.0-1.0)	0.0 (0.0-0.0)	<0.001
Use of antiemetics at PACU	10 (24.4)	1 (2.6)	0.012
Length of PACU stay (min)	40.0 (30.0-50.0)	40.0 (32.5-52.5)	0.428

Data are presented as median (interquartile range), or number of patients (%). NRS, an 11-point numeric rating scale (0 = no pain, 10 = worst imaginable pain); TIVA, total intravenous anesthesia; PACU, postanesthesia care unit; PONV, postoperative nausea and vomiting. *Emergence is defined as the period from the end of surgery to 2 min after extubation. †Time to verbal response is defined as the time from cessation of anesthetics to patients' response to verbal command. ‡Time to extubation is defined as the time from cessation of anesthetics to tracheal extubation. ¶Emergence agitation is defined as a sedation-agitation scale score of ≥ 5. §Grade of cough: 0, no cough; 1, single cough; 2, cough persistence ≥ 5 s; 3, persistent cough for ≥ 5 s or bucking. **Postoperative nausea and vomiting on a scale 0-3 (none, mild, moderate, and severe).

**Table 5:** Postoperative complications and hospital course

	Sevoflurane group (n = 41)	TIVA group (n = 39)	*P*
**Complications**			
SIADH	0 (0.0)	0 (0.0)	-
Transient diabetes insipidus	9 (22.0)	8 (21.1)	>0.999
Permanent diabetes insipidus	0 (0.0)	1 (2.6)	0.969
Infection	1 (2.4)	1 (2.6)	>0.999
Postop CSF leak	0 (0.0)	2 (5.3)	0.441
Epistaxis	4 (9.8)	6 (15.8)	0.640
Visual field defect	0 (0.0)	3 (7.9)	0.213
Reoperation within postoperative 30 days	0 (0.0)	3 (7.9)	0.213
Length of hospital stay	5.0 (5.0-6.0)	5.5 (5.0-8.0)	0.418

Data are presented as median (interquartile range), or number of patients (%). CSF, cerebrospinal fluid; SIADH, syndrome of inappropriate secretion of antidiuretic hormone; TIVA, total intravenous anesthesia.
